# Finite element analysis of the effect of residual lateral wall volume on postoperative stability in intertrochanteric fractures

**DOI:** 10.1186/s13018-023-04501-1

**Published:** 2024-01-20

**Authors:** Yachun Zhang, Enzhe Zhao, Jian Zhu, Dou Wu, Yujie Fu, Xingyu Zhang, Xiaolun Zhang, Xubin Song

**Affiliations:** Third Hospital of Shanxi Medical University, Shanxi Bethune Hospital, Shanxi Academy of Medical Sciences, Tongji Shanxi Hospital, Taiyuan, 030032 China

**Keywords:** Intertrochanteric fracture, Lateral wall fracture, Residual lateral wall volume, Finite element analysis

## Abstract

**Background:**

Lateral wall fractures represent crucial risk factors for postoperative internal fixation failure in intertrochanteric femoral fractures. However, no consensus exists on the type of lateral wall fracture requiring interventional management. This study aimed to investigate the effect of residual lateral wall volume on the postoperative stability of intertrochanteric femur fractures with associated lateral wall fractures, providing valuable reference for the clinical management of the lateral wall.

**Methods:**

Eleven bone defect models of intertrochanteric femur fractures with varying residual lateral wall volumes were constructed using finite element analysis. These models were fixed with proximal femoral nail antirotation (PFNA). Simulations of von Mises stress and displacement distribution of the PFNA and femur during normal walking were conducted. Statistical analysis was performed to assess the correlation between volume and the maximum von Mises stresses and displacements of the PFNA and femur.

**Results:**

In all 11 models, the maximum von Mises stress and displacement of the helical blade, intramedullary nail, and femur occurred at the same locations. As residual lateral wall volume increased, the maximum von Mises stress and displacement of the helical blade, intramedullary nail, and maximum femoral displacement gradually decreased. However, the overall trend of the maximum femoral von Mises stress gradually decreased. At 70% retention of the residual lateral wall volume, there was a more pronounced change in the value of the maximum stress change of the helical blade and the intramedullary nail. Statistical analysis, including the Shapiro–Wilk test and Pearson correlation analysis, demonstrated a significant negative correlation between volume and the maximum von Mises stress and displacement of the helical blade, intramedullary nail, and femur. Linear regression analysis further confirmed this significant negative correlation.

**Conclusion:**

Finite element analysis of the residual lateral wall revealed a significant correlation between volume and the postoperative stability of intertrochanteric femur fractures. A volume of 70% may serve as the threshold for stabilizing the residual lateral wall. Volume emerges as a novel index for evaluating the strength of the residual lateral walls.

## Background

Hip fractures are predominantly observed in the elderly population and are expected to account for 6.26 million cases by 2050 worldwide. Intertrochanteric fractures account for approximately 50% of hip fractures [1]. The integrity of the lateral wall plays a pivotal role in the management of femoral intertrochanteric fractures [2]. This component offers crucial support for the head and neck fracture block, facilitating lateral sliding in the direction of the cephalomedullary nail, promoting contact insertion of the fracture section, and fostering optimal fracture healing [3]. Conversely, when the lateral wall is compromised, support for the head and neck fracture block diminishes. This results in lateral sliding of the femoral head and neck fracture block, medial movement of the femoral stem, and overall destabilization of the internal fixation structure, culminating in fixation failure [4, 5].

Currently, the recommended approach for treating intertrochanteric fractures concomitant with lateral wall fractures involves an intramedullary fixation system, exemplified by the proximal femoral nail antirotation (PFNA) [6–8]. Although the intramedullary nail within this fixation system functions as a “metal lateral wall,” its design lacks efficacy in providing robust fixation for lateral wall fractures and fails to ensure timely restoration of lateral wall stability. In cases of intertrochanteric femoral fractures, inadequate repositioning of the lateral wall block results in compromised bony support for internal fixation, delayed fracture healing, instability of intertrochanteric fractures, and subsequent fixation failure [9]. Conversely, the reduction and fixation of the lateral wall can reinstate its supportive role, enhancing the stability of intertrochanteric femoral fractures and contributing to a favorable prognosis. However, consensus regarding the specific type of lateral wall fracture necessitating intervention remains elusive.

In the context of the lateral wall fractures, the remaining portion, known as the residual lateral wall, assumes a supportive role, particularly in connection with the femoral stem. Research focused on the residual lateral wall aims to determine its capacity to provide support following a lateral wall fracture, thereby determining the necessity for lateral wall reconstruction. Give the three-dimensional structure of the lateral wall, the consideration of residual lateral wall volume allows for the integration of two-dimensional data (thickness, length, and width), offering a more comprehensive understanding of the real-world dynamics of the lateral wall. This study employs finite element analysis to construct various volumes of the residual lateral wall, analyze stress and displacement distribution in the internal fixation model post-intertrochanteric fracture, theoretically establish the influence of volume on lateral wall strength, and examine the correlation between lateral wall volume changes and postoperative stability in intertrochanteric femoral fractures. This approach introduces a novel index for assessing residual lateral wall strength, serving as a reference for lateral wall reconstruction.

## Materials and methods

### Finite element model establishment

A healthy Chinese male volunteer was recruited for this study (age: 36 years; weight: 70 kg; height: 170 cm). Radiographic examination revealed a normal femur without any signs of femoral disease or deformity. The femur underwent CT scanning, and the data were saved in Digital Imaging and Communications in Medicine format. The collected CT image data were input into Mimics 19.0 to construct 3D femur models. Surface errors, such as spikes and crosses, in the 3D femur model were corrected using Geomagic Studio 10.0. After rectifying the surface roughness, a 3D smooth solid model was developed and imported into Creo 7.0 software. Subsequently, a model of the intertrochanteric fracture was created using Creo 7.0. Subsequently, a model of the intertrochanteric fracture was created using Creo 7.0, with the fracture line irregularly curved with reference to A1.2 in the AO/OTA-2018 fractures classification for a two-part fracture [10].

**Fig. 1:**
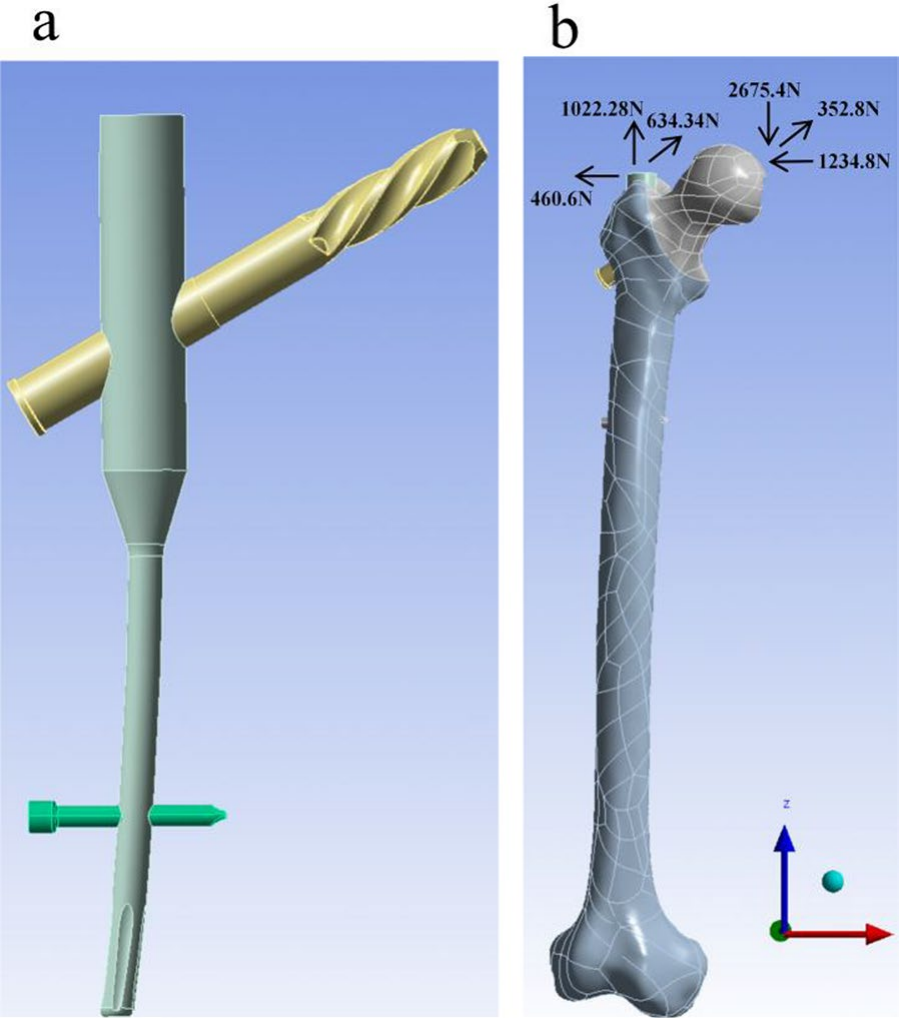
The PFNA model (**a**) and the model of femur and the loading acted on the model (**b**)

A 3D model of the PFNA was created using the Creo 7.0, with geometric dimensions obtained from the manufacturer’s catalog (Fig. 1a). The PFNA parameters were as follows: intramedullary nail length 170 mm, helical blade diameter 10 mm, length 100 mm, neck stem angle 130°, valgus angle 5°, and locking screw length 35 mm. The PFNA was assembled with the model of intertrochanteric fracture following surgical operation standards to ensure that the helical blade was located in the lower middle third of the femoral neck in the anteroposterior views and in the center of the femoral neck in the lateral views. The apical distance was < 25 mm.

**Fig. 2:**
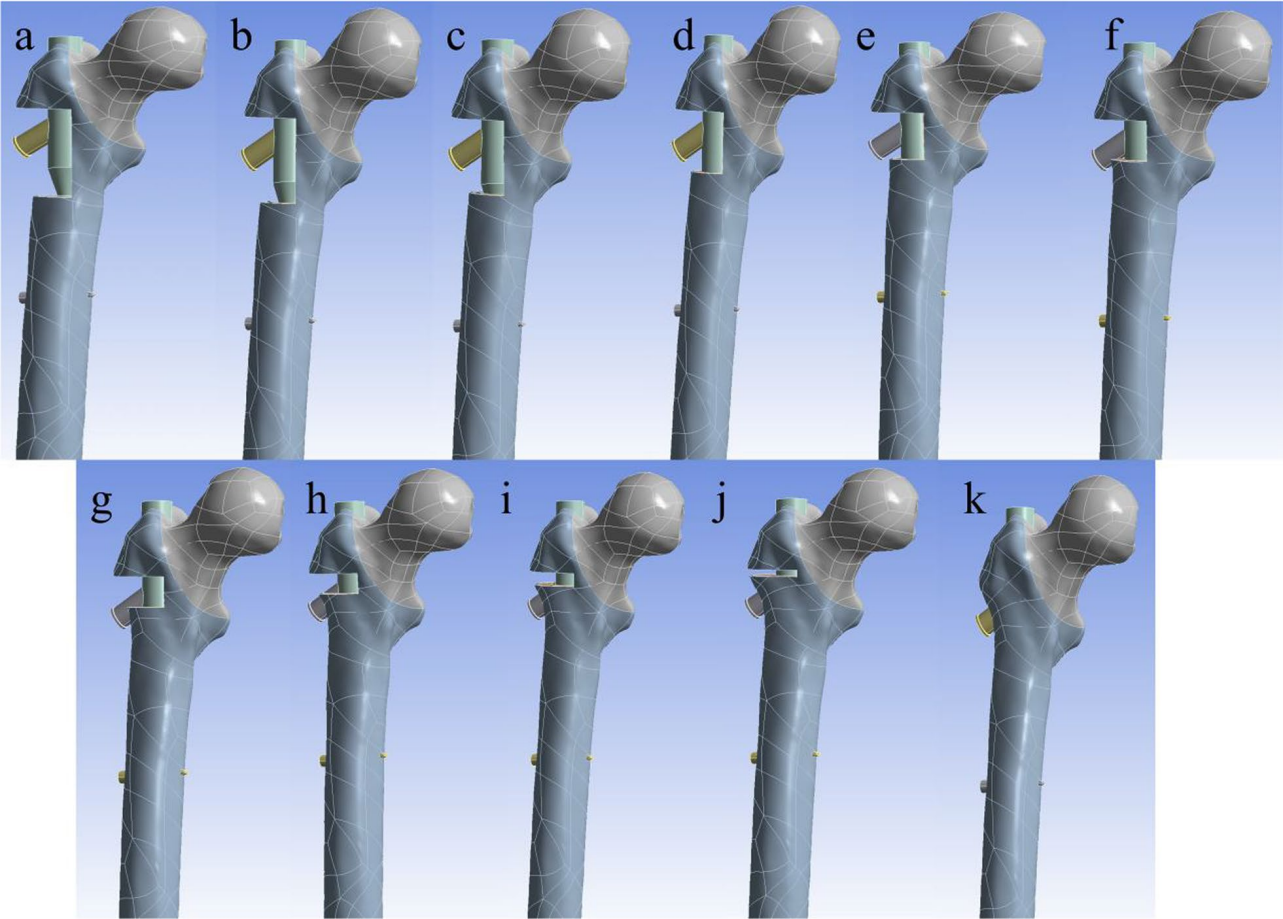
The **a**–**k** are models where the residual lateral wall volume is 0%, 10%, 20%, 30%, 40%, 50%, 60%, 70%, 80%, 90%, 100% of the volume of the lateral wall

Next, the bone defect in the lateral wall was modeled using Creo 7.0, and the extent of the bone defect in the lateral wall was defined with reference to the definition of Gao [11]. The upper boundary was the vastus lateralis ridge, and the lower boundary was the intersection of the tangent line of the cortex of the lower margin of the femoral neck and the lateral cortex of the femur. The defect depth was established as the cortex lateral to the femoral stem axis. Using Creo software version 7.0, the lateral wall was divided into 10 equal parts according to volume to construct 11 intertrochanteric femur fracture models with different volumes of lateral wall defects: 0%, 10%, 20%, 30%, 40%, 50%, 60%, 70%, 80%, 90%, and 100% of the total volume of the lateral wall volume in the residual lateral wall volume (Fig. 2).

Finally, the femur model with PFNA was imported into ANSYS Workbench 2022 R1 software for analysis, and the solid models were discretized into four-node tetrahedral cells using this software. Convergence tests were performed to determine the optimal maximum cell size for evaluating the accuracy of the finite element model. After convergence measurements, a mesh size of 2 mm was determined.

All bone and implant models were assumed to have homogeneous, isotropic, and linear elastic behaviors and were assigned the corresponding material properties based on the reported literature (Table 1). Frictional contact was used to characterize the contact interactions between bone fragments, implant components, bones, and implants. Referring to previous literature, the coefficient of friction is established at 0.46 between the fracture surfaces, 0.23 between implants, and 0.30 between bone and implants [12].

**Table 1:** Material properties used in the simulations in this study

Materia	Young’s modulus (Mpa)	Poisson’s ratio
Cortical bone	11,256	0.30
Cancellous bone	197.2	0.29
PFNA	11,000	0.33

### Boundary and loading conditions

For boundary conditions, the distal femur was constrained in all degrees of freedom [13]. The loading force acting on the femur represented the load at heel strike during normal walking. A joint reaction force of 2967.7 N ({*x*, *y*, *z*} = {− 1234.8, 352.8, − 2675.4}) was applied to the femur (4.2 times body weight). To reduce the bending moment of the proximal femur, an adductor load of 1288.3 N ({*x*, *y*, *z*} = {− 460.6, 634.34, 1022.28}) (1.9 times body weight) was applied at the greater trochanter [14] (Fig. 1b).

### Evaluation criteria

In the finite element analysis, the maximum von Mises stress and displacement were chosen as indicators of stability and internal fixation failure risk in PFNA-fixed intertrochanteric femoral fractures. The von Mises stresses and displacements of the PFNA and femur during normal walking were evaluated and analyzed for 11 residual lateral wall volume models. The maximum stress and displacement values of the helical blade, intramedullary nail, and femur were determined for each model group.

### Statistical analysis

SPSS 27.0 software was utilized for statistical analysis. The correlation between the volume of the residual lateral wall and the maximum von Mises stress and displacement of the helical blade, intramedullary nail, and femur was assessed using the Shapiro–Wilk test for conformity for normal distribution conformity in each data group. Pearson’s correlation analysis was employed if the data conformed to normal distribution, whereas Spearman’s correlation analysis was used if it did not. Linear regression analysis was performed to further validate the correlation between the volume, maximum von Mises stress, and displacement of helical blade, intramedullary nail, and femur. A *p* < 0.05 was considered statistically significant.

## Results

### Von Mises stress distribution of the PFNA

**Fig. 3:**
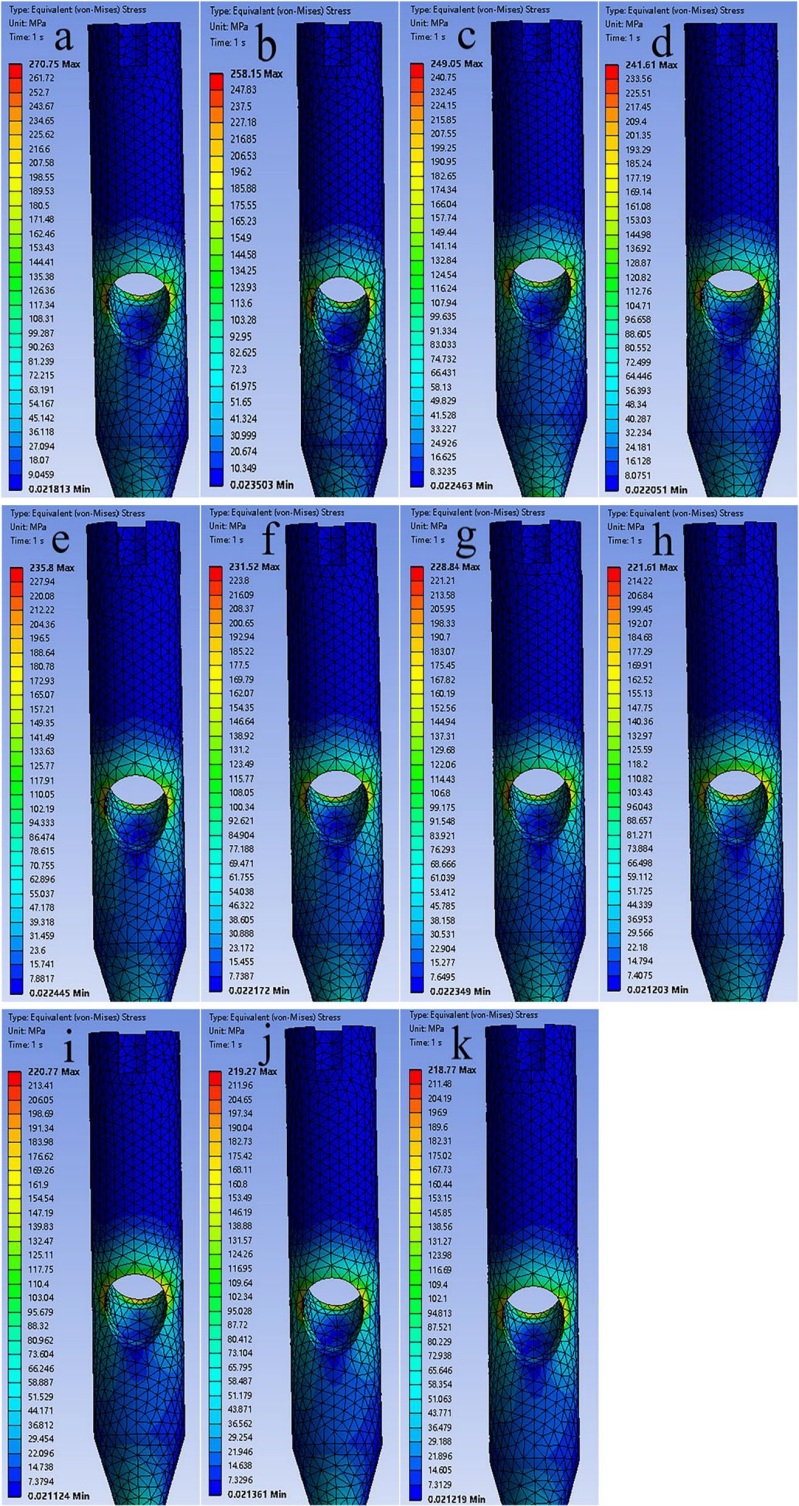
The **a**–**k** are the von Mises stress distributions of the intramedullary nails for 11 models from 0 to 100%

**Fig. 4:**
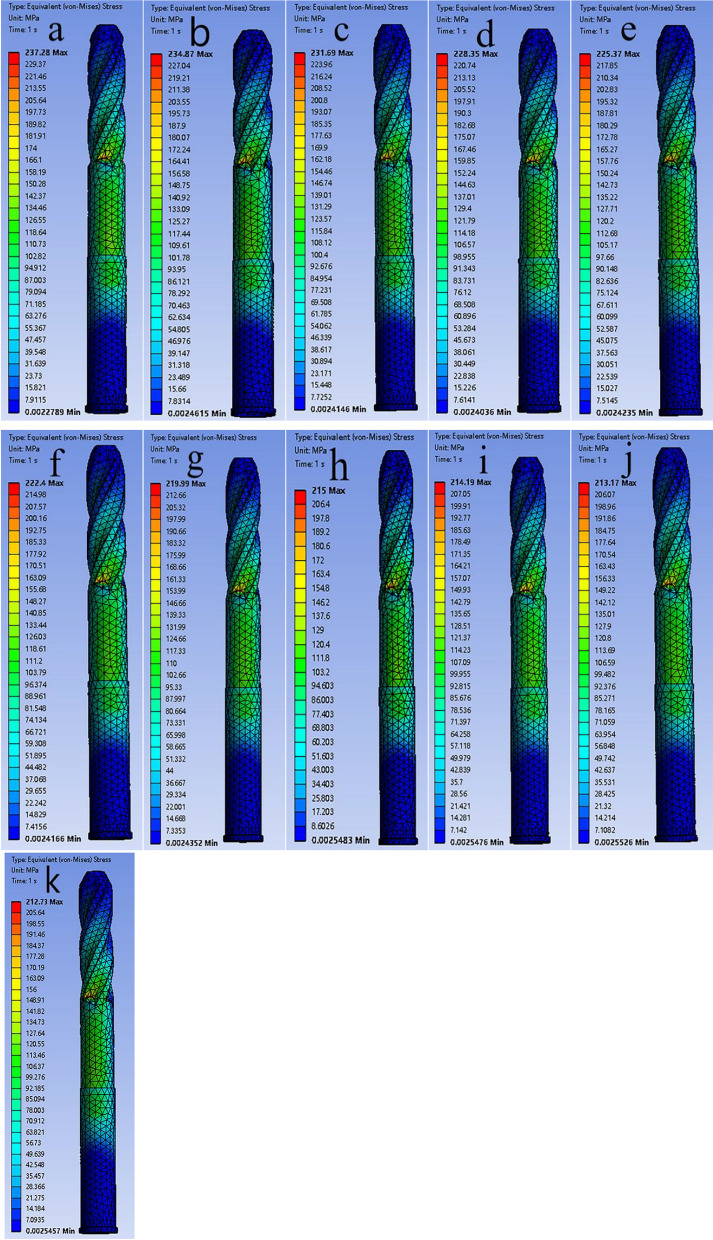
The **a**–**k** are the von Mises stress distributions of the helical blades for 11 models from 0 to 100%

**Fig. 5:**
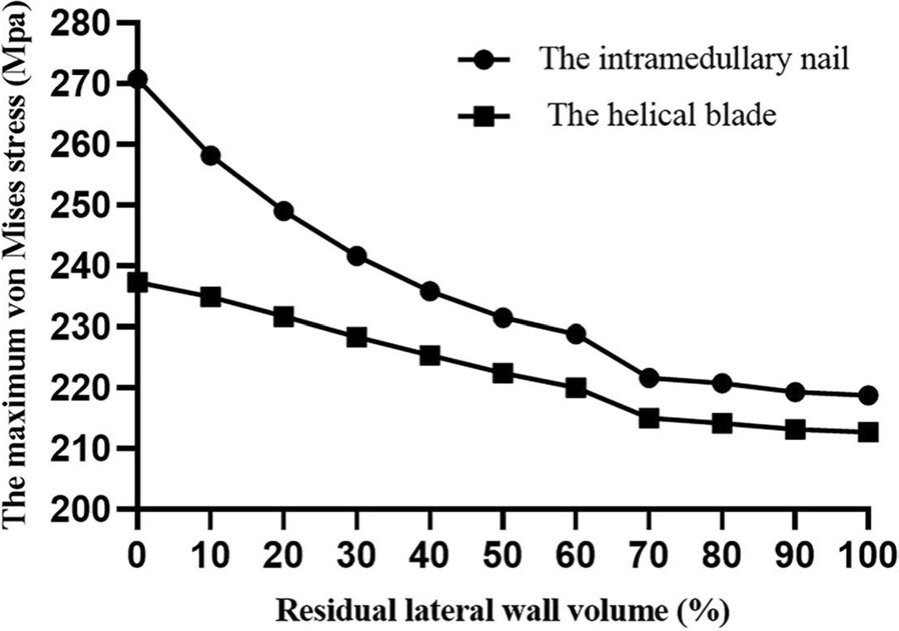
Volume versus maximum von Mises stress for helical blades and intramedullary nails

The maximum von Mises stresses in the helical blades and intramedullary nails across all the 11 models were observed at the same location. The maximum von Mises stresses of the intramedullary nails in the 11 models with 0–100% volume were 270.75 Mpa, 258.15 Mpa, 249.05 Mpa, 241.61 Mpa, 235.80 Mpa, 231.52 Mpa, 228.84 Mpa, 221.61 Mpa, 220.77 Mpa, 219.27 Mpa, 218.77 Mpa (Fig. 3). The maximum von Mises stresses of the helical blades in the 11 models were 237.28 Mpa, 234.87 Mpa, 231.69 Mpa, 228.35 Mpa, 225.37 Mpa, 222.40 Mpa, 219.99 Mpa, 215.00 Mpa, 214.19 Mpa, 213.17 Mpa, and 212.73 Mpa (Fig. 4). Given that the volume of the residual lateral wall increases, the maximum stress on the helical blade and intramedullary nail gradually decreases. The values of maximum stress changes in the helical blade and intramedullary nail were large at 0–70% of the volume. And the values of changes after 70% were small (Fig. 5).

### Von Mises stress distribution of the femur

The maximum von Mises stress in the femur was consistently located at the same location across all the 11 models. The overall trend revealed a gradual decrease in the maximum femoral von Mises as the volume of the residual lateral wall increased.

### PFNA and femur displacement distribution

**Fig. 6:**
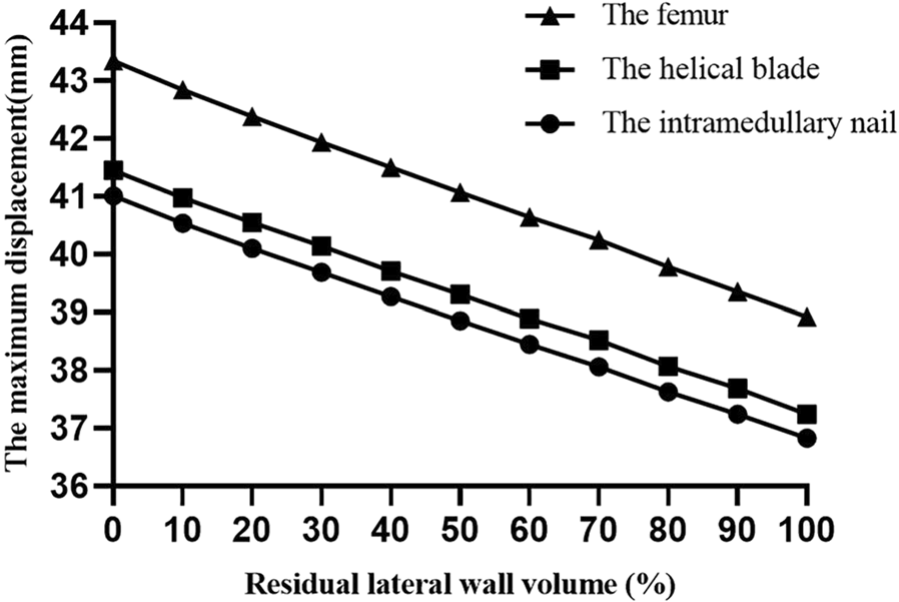
Volume versus maximum displacement of intramedullary nails, helical blades, and femurs

**Fig. 7:**
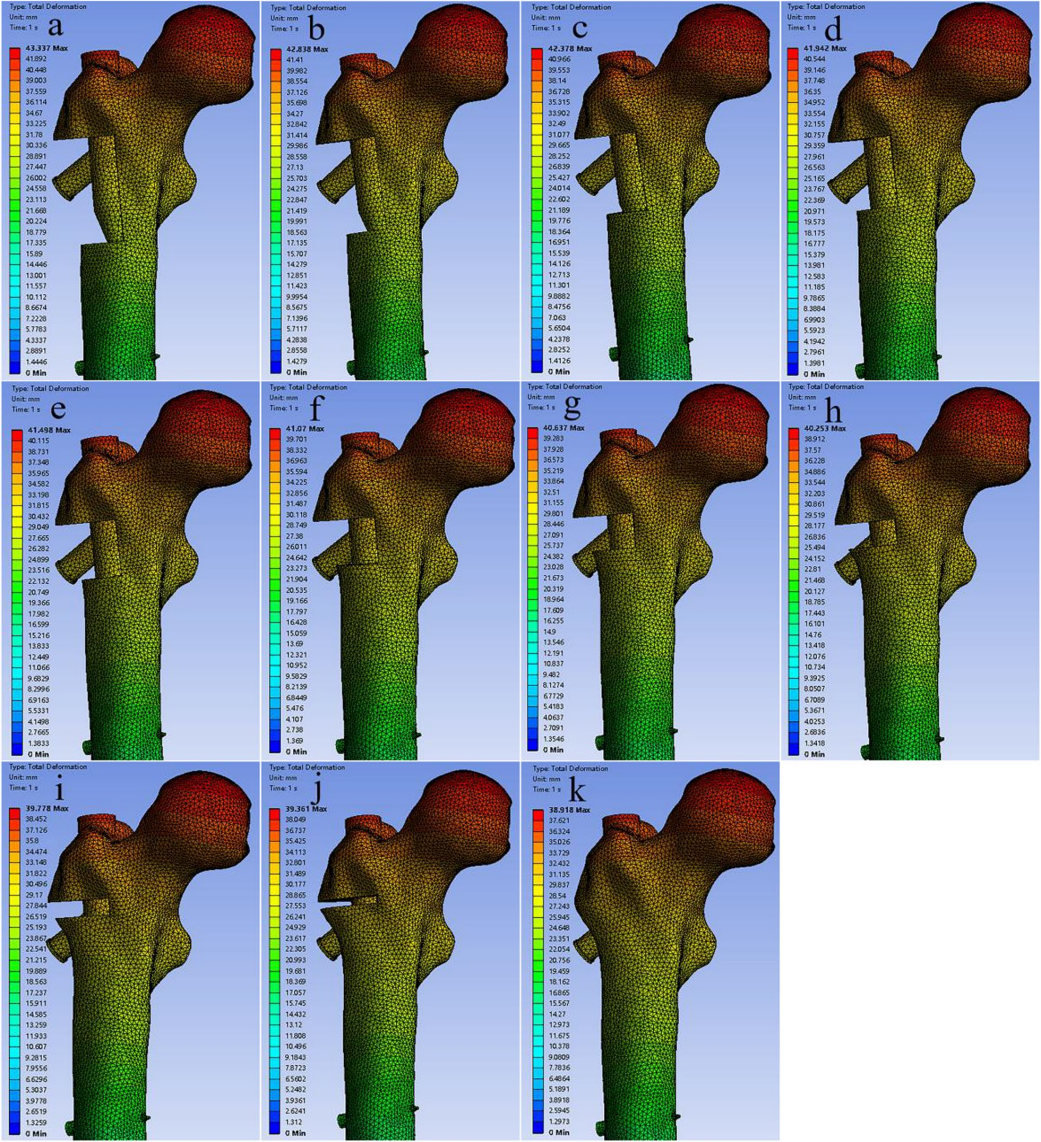
The **a**–**k** are the displacement distributions of the femur for 11 models from 0 to 100%

The maximum displacements of the helical blade, intramedullary nail, and femur occurred at identical locations in all 11 models. As the residual lateral wall volume increased, the maximum displacements of the helical blade, intramedullary nail, and femur exhibited a gradual decreased (Fig. 6). Specifically, the femoral displacements from 0 to 100% of the volume were 43.337 mm, 42.838 mm, 42.378 mm, 41.942 mm, 41.498 mm, 41.070 mm, 40.637 mm, 40.253 mm, 39.778 mm, 39.361 mm, and 38.918 mm, respectively (Fig. 7).

### Volume correlation analysis with maximum von Mises stress and displacement of PFNA and femur

The data from each group conformed to a normal distribution using the Shapiro–Wilk test and were analyzed using Pearson correlation. The results revealed a significant negative correlation between volume and maximum von Mises stress and displacement of the helical blade, intramedullary nail, and femur (*P* < 0.05). The absolute values of the correlation coefficients (|*r*|) between the volume and maximum von Mises stress and displacement of the helical blade, intramedullary nail, and maximum displacement of the femur were all > 0.8, indicating a strong correlation (Table 2). The correlation between volume and each data group was further verified using linear regression analysis, which also demonstrated that volume was significantly negatively correlated with the maximum von Mises stress and displacement of the helical blade, intramedullary nail, and femur (Table 3).

**Table 2:** Correlation analysis of volume with maximum von Mises stress and displacement of PFNA and femur

Parameters	*r*	*P*
The maximum von Mises stress of the intramedullary nail(Mpa)	− 0.955	< 0.001
The maximum displacement of the intramedullary nail(mm)	− 1	< 0.001
The maximum von Mises stress of the helical blade(Mpa)	− 0.987	< 0.001
The maximum displacement of the helical blade(mm)	− 1	< 0.001
The maximum von Mises stress of the femur (Mpa)	− 0.696	< 0.05
The maximum displacement of the femur (mm)	− 1	< 0.001

**Table 3:** Results of linear regression analysis of volume versus PFNA and femoral maximum von Mises stress and displacement

Parameters	Model	B	Beta	t	95% CI	*P*
The maximum von Mises stress of the intramedullary nail (Mpa)	Constant	260.87		85.26	(253.96, 267.81)	< 0.001
	Volume (%)	− 49.75	− 0.96	− 9.62	(− 61.45, − 38.05)	
The maximum displacement of the intramedullary nail (mm)	Constant	40.95		2718.86	(40.92, 40.99)	< 0.001
	Volume (%)	− 4.15	− 1.00	− 162.79	(− 4.20, − 4.09)	
The maximum von Mises stress of the helical blade (Mpa)	Constant	236.56		275.57	(234.61, 238.50)	< 0.001
	Volume (%)	− 26.74	− 0.99	− 18.43	(− 30.02, − 23.46)	
The maximum displacement of the helical blade (mm)	Constant	41.40		2834.22	(41.37, 41.43)	< 0.001
	Volume (%)	− 4.16	− 1.00	− 168.30	(− 4.21, − 4.10)	
The maximum von Mises stress of the femur (Mpa)	Constant	454.33		64.48	(438.39, 470.27)	< 0.05
	Volume (%)	− 34.63	− 0.70	− 2.91	(− 61.57, − 7.69)	
The maximum displacement of the femur (mm)	Constant	43.28		2546.86	(43.240, 43.32)	< 0.001
	Volume (%)	− 4.37	− 1.00	− 152.11	(− 4.43, − 4.30)	

## Discussion

The concept of the lateral femoral wall, as introduced by Gotfried in 2014, defined the lateral cortex of the proximal femur, where the cephalomedullary nail is driven, as the lateral wall [15]. Hsu, in 2013, proposed the concept and measurement of lateral wall thickness, establishing that a thickness of < 20.5 mm predisposes to perioperative lateral wall fractures [16]. This notion was incorporated into the AO/OTA-2018 fractures classification, where lateral wall status became the basis for staging A1 and A2, using a lateral wall thickness of 20.5 mm as the threshold for both types, thus emphasizing the importance of the lateral wall in intertrochanteric fractures and elevating its importance [17].

Most existing research on the lateral wall has primarily focused on predicting the risk of perioperative lateral wall fracture. However, managing already existing lateral wall fractures remains a topic of debate. Currently, femoral intertrochanteric fractures combined with lateral wall fractures are commonly treated with intramedullary fixation, exemplified by PFNA [18–20]. Despite its prevalence, this method fails to effectively fix lateral wall fractures and restore lateral wall stability promptly, resulting in compromised fracture restoration, decreased stability, and eventual internal fixation failure. Lateral wall fractures are frequently encountered in clinical practice, and recent in-depth studies have underscored their role in fixation failure [21]. Gao et al. [11] analyzed 821 intertrochanteric femur fractures, revealing that 12.1% were combined with lateral wall fractures. The failure rate of intramedullary fixation in simple intertrochanteric femoral fractures was 1.66%, whereas the rate surged to 11.1% when combined with lateral wall fractures. Fan et al. [22] found a failure rate of 7.69% in 130 intertrochanteric femoral fractures combined with lateral wall fractures using intramedullary fixation. Several scholars have explored lateral wall reconstruction, noting its favorable outcomes. Kulkarni et al. [23], for instance, reconstructed the lateral wall using cerclage wire and lag screws, resulting in significantly lower rates of postoperative hip varus malunion and fracture nonunion. Wang et al. [24] employed a lateral wall reconstruction plate combined with PFNA, demonstrating its superiority over intramedullary nailing alone in terms of fracture healing time, internal fixation failure complication rate, and postoperative functional recovery. In elderly patients with osteoporosis, bone cement can be used jointly to strengthen the helical blade to enhance the stability of the bone–screw interface and increase the ability of the cancellous bone to carry stress and enhance the stability of the fracture end, which can lead to rapid stabilization of the fracture end, reduce pain in the affected limb, and facilitate functional exercise as early as possible [25].

Despite these advancements, there remains no consensus on the types of lateral wall fractures that necessitate reconstruction. Chang et al. [26] measured the residual lateral wall width in the plane of the midpoint of the lesser trochanter on 3D reconstructed CT in 55 cases of intertrochanteric fractures with combined lateral wall fracture using PFNA. The goal was to assess whether the fracture line in the coronal plane involved the entry point of the helical blade. They found that the likelihood of postoperative complications was significantly higher in the group with a rupture at the entry point, suggesting that a residual lateral wall width of ≤ 18.55 mm is a crucial factor in predicting the risk of a rupture at the entry point. A residual lateral wall width of ≤ 18.55 mm was identified as an important predictor of nail entry point rupture. Thus, the residual lateral wall width may serve as an indicator of postoperative stability in intertrochanteric femoral fractures combined with lateral wall fractures.

When the lateral wall is fractured, it does not imply completely dysfunction; the residual lateral wall may continue to fulfill its supportive function. However, when the lateral wall breaks to a certain extent, its function is entirely lost, and clinical intervention may be required. In this study, the residual lateral wall volume was set as the only variable to ensure accuracy of the experimental results. On the one hand, the fracture line was uniformly set to type A1.2 to avoid the influence of the fracture type on postoperative stability. On the other hand, the femoral and PFNA devices were assembled in strict accordance with the manufacturer’s instructions to exclude the influence of the PFNA device position on the test. Bartoska et al. [27] found that absolute precision of positioning was not strictly required for stable intertrochanteric fractures and that small deflections in the screw position did not significantly increase the overall risk of fixation failure. Intertrochanteric fractures combined with lateral wall fractures are often unstable, and the placement of the screw blade in an appropriate position in the femoral neck may result in greater stability after fracture reduction [28]. In this study, the helical blade was orthogonally located in the middle and lower 1/3 of the femoral neck and laterally located in the center of the femoral neck so that the helical blade was close to the femoral calcar to obtain greater axial and torsional stiffness. The extent of the lateral wall was delineated on 3D reconstructed CT with reference to the definition of Gao et al. [11]. The upper boundary, the lateral femoral muscular crest, is an easily recognizable anatomical landmark at the junction of the proximal cortical and cancellous bones. A fracture below the lower boundary, below the intersection of the tangent line of the inferior border of the femoral neck and lateral cortical femur, is a subtrochanteric fracture. This region is the primary region where the head and neck nails are driven, all of which are cortical and supportive, and the definition of this range is more clinically relevant.

Using finite element analysis, we found that an intact lateral wall plays an important role in the postoperative stability of intertrochanteric fractures. When the lateral wall was intact, that is, 100% of the volume of the residual lateral wall, the maximum von Mises stresses of the helical blade and intramedullary nail were 212.73 Mpa and 218.77 Mpa, respectively. When the lateral wall was completely missing, that is, 0% of the volume, the maximum von Mises stresses of the helical blade and intramedullary nail increased by 11.5% and 23.8% to 218.77 Mpa and 212.73 Mpa, respectively. As the residual lateral wall volume increased, the maximum von Mises stress and displacement of the helical blade, intramedullary nail, and femur decreased, indicating increased postoperative stability of the intertrochanteric fractures. From the changes in the maximum von Mises stress values of the helical blade and intramedullary nail, the magnitude of the von Mises stress gradually decreased with the integrity of the volume. At 70% volume retention, the magnitude of the von Mises stress changes exhibited a more pronounced alteration, with a relatively substantial magnitude of von Mises stress changes before 70%, following which the von Mises stress changes gradually stabilized. The maximum von Mises stress in the helical blade decreased by 12.6 Mpa from 100 to 90% of the residual lateral wall volume. The volume decreased by 9.1 Mpa, 7.44 Mpa, 5.81 Mpa, 4.28 Mpa, 2.86 Mpa, and 7.23 Mpa for every 10% from 90 to 70%. When increasing from 70 to 100%, decrease of 0.84 Mpa, 1.5 Mpa, 0.5 Mpa, respectively. The maximum von Mises stresses of the same intramedullary nail were reduced by 2.41 Mpa, 3.18 Mpa, 3.34 Mpa, 2.98 Mpa, 2.97 Mpa, 2.04 Mpa, 5 Mpa for every 10%, from 100 to 70%, and from 70 to 100% at 0.81 Mpa, 1.02 Mpa, 0.44 Mpa. From 70 to 100%, the maximum von Mises stresses in the helical blade and intramedullary nail were reduced by 1.0% and 1.2%, respectively. Therefore, we hypothesized that maintaining a residual outer lateral wall volume of 70% may serve as the threshold for the residual outer lateral wall to fulfill its function. When the lateral wall volume is > 70%, it offers support to the head, neck fracture block, and internal fixation device, contributing to the enhanced stability of intertrochanteric fractures. Conversely, when the volume is < 70%, the residual lateral wall loses its supportive function and stability. In such cases, intervention in lateral wall treatment, such as reconstruction, may be required, necessitating a delay in weight-bearing.

This study has several limitations. The femurs and implants, characterized by anisotropic materials, they were simplified as homogeneous, isotropic, and elastic materials to streamline the analysis. However, this study was not validated experimentally, which is a common limitation of similar simulation studies. The research team used an artificial femur for biomechanical analysis to validate the conclusions of this study and obtained similar findings. Our research team is currently attempting to obtain biomechanical analysis findings and validate them in a clinic to guide clinical diagnosis and treatment.

## Conclusion

This biomechanical analysis delved into intertrochanteric femoral rotor fractures with varying residual lateral wall volumes stabilized using PFNA. The study conclusively revealed a close association between volume indicators and the postoperative stability of intertrochanteric fractures. Notably, a volume of 70% emerged as a potentially critical threshold for the residual lateral wall to effectively function as a stabilizer. The incorporation of residual lateral wall volume holds promise for clinicians in assessing lateral wall strength, offering valuable insights for decisions related to lateral wall reconstruction and determining weight-bearing timelines.

## Data Availability

Please contact author for data requests.

## Abbreviations

PFNA: Proximal femoral nail antirotation
CT: Computed tomography
3D: Three dimensional
